# Numerical Relationships Between Archaeal and Bacterial *amoA* Genes Vary by Icelandic Andosol Classes

**DOI:** 10.1007/s00248-017-1032-9

**Published:** 2017-07-13

**Authors:** Hendrikus J. Laanbroek, Peter T. M. Veenhuizen, Rosalinde M. Keijzer, Mariet M. Hefting

**Affiliations:** 1 Department of Microbial Ecology, Netherlands Institute of Ecology (NIOO-KNAW), P.O. Box 50, 6700 AB, Wageningen, the Netherlands; 2 Ecology and Biodiversity Group, Department of Biology, Utrecht University, Utrecht, the Netherlands

**Keywords:** Ammonia oxidation, *amoA* gene, Archaea, Bacteria, Andosols, Iceland

## Abstract

Bacterial *amoA* genes had not been detectable by qPCR in freshly sampled Icelandic Andosols thus far. Hence, a new primer set yielding shorter gene fragments has been designed to verify the absence of ammonia-oxidizing bacteria in different Icelandic Andosol classes. At the same time, a new primer set was also constructed for archaeal *amoA* genes that should improve the quality of PCR products. Although a large part of the soil samples were found to be *amoA*-negative, bacterial *amoA* genes were detectable with new as well as old primer sets. The same results were obtained for the archaeal *amoA* genes. The relative distribution of archaeal and bacterial *amoA* genes varied between Andosol classes. Archaeal *amoA* genes were significantly more abundant in Brown than in Histic Andosols, while the opposite was observed for bacterial *amoA* genes. The numbers of archaeal and bacterial *amoA* genes in Gleyic Andosols were not significantly different from those in Histic and Brown Andosols. The numbers of bacterial *amoA* genes, but not the numbers of archaeal *amoA* genes, correlated significantly and positively with potential ammonia oxidation activities. The presence of the bacterial nitrification inhibitor allylthiourea inhibited the potential ammonia oxidation activities during the first 12 h of incubation. Hence, it was concluded that ammonia-oxidizing bacteria profited most from the conditions during the measurements of potential ammonia oxidation activities.

**Electronic supplementary material:**

The online version of this article (doi:10.1007/s00248-017-1032-9) contains supplementary material, which is available to authorized users.

## Introduction

Aerobic ammonia- and nitrite-oxidizing microorganisms are indispensable for a well-functioning nitrogen cycle as they are the only link between its most reduced and oxidized parts. At the same time, the process of aerobic ammonia oxidation is the basis of many environmental problems such as pollution of ground and surface waters with the mobile nitrate ion, acidification, and subsequent weathering of soils and rocks, and emission of the potent greenhouse gas nitrous oxide. Hence, there are many arguments to study the behavior of aerobic ammonia-oxidizing microorganisms in the environment. Aerobic ammonia-oxidizing microorganisms belong to two evolutionary domains of life, i.e., the Archaea and the Bacteria. Ammonia-oxidizing bacteria have been known since the late nineteenth century and have since then been isolated from different environments as summarized by Koops et al. [23]. All the isolated ammonia-oxidizing bacteria oxidize ammonia by the enzyme ammonia monooxygenase, which is encoded by different genes in the *amo* operon. Ammonia-oxidizing archaea have only been known a little more than a decade by the discovery of *amoA* genes that were homologs to bacterial *amoA* genes but were clearly from archaeal origin [41, 42]. The ability of archaea to oxidize ammonia to nitrite has since then been shown in isolated pure cultures of ammonia-oxidizing archaea [22, 40]. Even more recently, a complete set of genes involved in ammonia oxidation have been detected in nitrite-oxidizing *Nitrospira* species [12, 20, 29] expanding the group of potentially ammonia-oxidizing microorganisms in natural and man-made ecosystems.

Since the discovery of ammonia-oxidizing archaea, many studies were initiated to look for differences in distribution of both groups of ammonia-oxidizing microorganisms in natural habitats. The first study of relative abundances of ammonia-oxidizing archaea and bacteria in soils was performed by Leininger et al. [26]. In 12 soils differing in texture and management histories, archaeal *amoA* gene copies outnumbered the bacterial *amoA* gene copies suggesting a significant role of archaea in ammonia oxidation in these soils, although growth strategies other than ammonia oxidation cannot be excluded based on more recent knowledge. Stopnišek and colleagues studying the effect of ammonium additions on *amoA* gene abundance in acidic, organic forest soils did not observe an effect of such additions on the abundance of archaeal *amoA* gene copies and suggested that the *amoA*-containing archaea may actually generate their energy for growth from the oxidation of organic nitrogen compounds [37]. Levicnik-Höfferle and colleagues observed a positive response of archaeal *amoA* gene abundance in acidic, organic marshland soil to the addition of organic nitrogen compounds but not to the addition of ammonium or urea [27]. These and other studies led to the idea of niche differentiation between ammonia-oxidizing archaea and bacteria [16, 30]. Environmental factors that might favor ammonia-oxidizing archaea over their bacterial counterparts are low ammonium concentration, the presence of small organic molecules, low pH, oxygen limitation, and elevated temperatures. Stress provoked by one or by combinations of these factors may lead to the apparent absence of ammonia-oxidizing bacteria in certain soils. In a survey that aimed at establishing drivers of ammonia-oxidizing communities, bacterial *amoA* genes were below the detection limit in 62.4% of 710 Scottish soil samples examined [48]. In contrast, archaeal *amoA* genes were not detected in 20.5% of the Scottish soil samples studied demonstrating that archaeal ammonia-oxidizing archaea can be successful where ammonia-oxidizing bacteria cannot. Whereas archaeal and bacterial *amoA* genes are usually found together in soils [14, 21, 25, 26, 28, 39, 49], acidic, organic soils disclose often only archaeal *amoA* genes [27, 37, 48]. So surprisingly, absence of bacterial *amoA* gene copies was also observed in near neutral grassland soils from Grændalur valley on Iceland [8]. Bacterial *amoA* gene copies came only to the fore after incubation of these soils in microcosms in the presence of ammonium [8, 10]. As these soils were classified as Histic Andosols [2, 3], it was at that time concluded that a high cation exchange capacity that commonly occurs in alkaline Andosols [44] caused apparently a strong binding of ammonium to soil particles and thereby repressing the abundance of bacterial ammonia oxidizers in these soils. Andosols cover a large part of Iceland and are subdivided in Histic, Gleyic, and Brown Andosols mainly on the basis of their moisture and carbon contents [2, 3]. Histic Andosols have a larger amount of organic carbon and soil moisture than consecutively Gleyic and Brown Andosols. The presence of organic carbon compounds in Histic Andosols will likely lead to increased nitrogen mineralization during warm and dry periods of the year. In a ^15^N-tracing experiment with samples collected from Icelandic grassland soils of ambient temperature, Daebeler et al. observed a significant and positive effect of incubation temperature on gross nitrogen mineralization and nitrification rates during an incubation period of 6 days [11]. This showed that the nitrogen cycle in these soils was sensitive to temperature changes. When Histic Andosols with periods of nitrogen mineralization are unable to maintain a community of bacterial ammonia oxidizers, then certainly the even more nitrogen-limited Gleyic and Brown Andosols will lack such a community. Hence, we hypothesized that bacterial ammonia oxidizers are below the detection limit in Icelandic Andosols and archaeal ammonia oxidizers will always prevail in these soils.

To test this hypothesis, archaeal and bacterial *amoA* genes were enumerated by qPCR and by measuring the potential ammonia oxidation activities in the absence and presence of allylthiourea (ATU), which is a known inhibitor of bacterial ammonia oxidation [25, 34, 38, 39], although it may also inhibit ammonia-oxidizing archaea partially [17, 25, 33, 34].

## Materials and Methods

### Sampling Sites

At the end of May 2013, soil samples have been collected in grass and heathlands at seven different locations in the southwestern part of Iceland. The choice of the sampling locations was based on the Icelandic soil map 1: 500,000 (http://k-sql.lbhi.is/desert/2-1.html) combined with field characteristics (i.e., estimated carbon content) (Supplementary Table S1). Based on measured moisture and carbon contents, the collected soils were characterized as Histic, Gleyic, and Brown Andosols according to Arnalds and Gretarsson [3]. One of the Histic Andosols (i.e., location 1) was one of the locations sampled by Daebeler et al. in August 2010 [8]. At each location, five individual samples have been collected from the top 10 cm of the soils leading to 35 samples in total. Upon arriving in the field laboratory, the individual samples were separately mixed, stripped of larger gravel particles and roots, and kept in 50 ml polypropylene screw cap tubes at 4 °C until the analyses started at the Netherlands Institute of Ecology (NIOO-KNAW) Wageningen, the Netherlands. Upon arrival, a subsample of the mixed soil samples was freeze-dried for determination of total soil carbon and nitrogen, and for molecular-genetic analyses.

### Analyses of Abiotic Soil Factors

For the determination of soil moisture content, 10 g of fresh subsample was weighted and reweighted after 48 h of drying at 70 °C. Soil pH-H_2_O was determined in soil slurries of 10 g fresh soil in 50 ml water. These slurries had been shaken at room temperature for 1 h. Freeze-dried soil samples were ground at 20 rpm using a ball mill (Retsch GmbH, Haan, Germany) and subsequently used for the determination of total C and total N by means of an elemental CN analyzer (InterScience BV, Breda, the Netherlands). Amounts of mineral nitrogen were determined in soil extracts obtained after shaking 10 g of soil in 50 ml of 1 M KCl for 1 h. The extracts were analyzed with a Quaatro Seal autoanalyzer (Beun—De Ronde, Abcoude, the Netherlands).

### DNA Extraction from Soils

Total genomic DNA was extracted by using 0.5 g of freeze-dried sample that was homogenized in 1 ml cetyltrimethylammonium bromide (CTAB) buffer in MP Lysing Matrix tubes (MP Biomedicals, Santa Ana, California, USA), subjected twice to disruption by bead-beating at a 5.0-m/s rotation for 60 s, and incubated at 37°C for 30 min in the presence of 5 μl proteinase K (20 mg/ml) while vortexing every 15 min. The samples were supplemented with 150 μl of 20% sodium dodecyl sulfate (SDS) solution, incubated at 65°C for 1 h in a thermoblock, and vortexed every 15 to 20 min. After centrifugation at 10,000×*g* for 10 min, approximately 500 μl of the supernatant was collected and directly added to the lysis buffer of the Maxwell®16 (Promega, Fitchburg, Wisconsin USA) DNA tissue extraction kit. Automatic purification of genomic DNA was performed using the tissue DNA extraction program.

### qPCR of Archaeal and Bacterial *amoA* Genes

Because of the inability of Daebeler et al. [8] to detect bacterial *amoA* genes in Icelandic Histic Andosols with a commonly used PCR primer set [31, 36], a new reversed primer for bacterial *amoA* genes was designed (Table 1). At the same time, a new reversed primer was constructed for the archaeal *amoA* gene. The new combinations of primers yield shorter gene fragments (110 versus 650 bp for the new and old archaeal primer set, respectively, and 175 versus 490 bp for the new and old bacterial primer set, respectively) and are therefore more dedicated to qPCR resulting in lower detection limits and better interpretable qPCR data. Conditions for the qPCR analyses of the archaeal *amoA* gene were an initial step of 10 min at 95 °C, 40 cycles of 15 s at 95 °C, 45 s at 58°C, and 45 s at 72°C. Conditions for the qPCR analyses of the bacterial *amoA* gene were an initial step of 10 min at 95 °C, 40 cycles of 15 s at 95 °C, 45 s at 56 °C, and 45 s at 72 °C. The qPCR runs were completed by creating a melting curve from 60 to 95 °C for quality control. The qPCR assays were performed in duplicate in reaction volumes of 10 μl containing Perfecta™ SYBR® Green SuperMixes (QuantaBio, Beverly, Massachusetts, USA), 0.21 mg/ml BSA (Roche, Cat. No. 10735078001), 0.25 μM forward and reverse primer (Integrated DNA Technology, Leuven, Belgium), and 2.5 μl DNA template. Reactions were performed in a ViiA7 real-time PCR instrument (Life Technologies, Bleiswijk, the Netherlands). To determine the quantity of the *amoA* genes in the DNA samples, cycle threshold (*C*_t_) values of the samples were compared with calibration lines made from plasmid DNA containing the archaeal or the bacterial *amoA* PCR fragment. A control reaction without template DNA was included in each qPCR assay. All DNA samples and the negative control were analyzed in duplicates to obtain an accurate value for the *amoA* gene abundance in each soil sample. The amplification efficiency calculated from the calibration lines ranged from 87 to 102% with *R* ^2^ values greater than 0.99. Six different PCR samples from both the old and the new primer combinations were checked by Sanger sequencing. To ensure sequence read without ambiguities, PCR products were inserted into a pCR2.1-TOPO vector (TOPO® TA cloning kit, Carlsbad, USA, Invitrogen). *E. coli* top 10 chemical competent cells were transformed with the new vector and grown on LB-ampicillin agar plates. Sequencing reactions on plasmid DNA from 30 single colonies were performed by Macrogen, Amsterdam, the Netherlands. Sequence identification was done by the BLASTN facility from the National Center for Biotechnology Information (http://www.ncbi.nlm.nih.gov/).

**Table 1:** Primers used in the qPCR of archaeal and bacterial *amoA* genes

Primer name	*amoA* target	DNA sequence	Ref.
Arch-amoAF	Archaeal	STAATGGTCTGGCTTAGACG	[15]
Arch-amoAR	Archaeal	GCGGCCATCCATCTGTATGT	[15]
AOA_amoA_175Brev	Archaeal	GTCCAiGCCCARTCiGTRTAGAA^a^	This article
amoA 1F	Bacterial	GGGGHTTYTACTGGTGGT	[36]
amoA 2R	Bacterial	CCCCTCKGSAAAGCCTTCTTC	[31]
AOB_amoA_405Crev	Bacterial	TGiGTiGGiCCRAAiATiGiCCAGTTRCC	This article

^a^ *i* = inosine

### Determination of Potential Ammonia Oxidation Activities

To estimate the numbers of active ammonia-oxidizing archaea and bacteria in each soil sample, potential ammonia oxidation activities were determined in slurries of 15 g fresh weight soil mixed with 50 ml of mineral medium containing ammonium at a final concentration of 1 mM, according to the protocol of Belser and Mays [5], as modified by Verhagen and Laanbroek [43]. Briefly, the linear production of nitrite plus nitrate over a time period of 72 h at in situ pH, and 20 °C is taken as a measure of the potential rate of ammonium oxidation. Samples from nitrite plus nitrate determinations were taken at 3, 6, 9, 12, 24, 48, and 72 h after addition of ammonium. In a parallel series of measurements, 100 μM ATU was added to the soil slurries to inhibit specifically the ammonia-oxidizing bacteria [25, 34, 38, 39]. Since five individual samples had been collected from each sampling location, the number of replicates per location was five.

### Statistical Analyses

All statistical analyses were performed with the IBM SPSS software package version 23 (IBM Corp. Armonk, NY). Before the analyses started, outliers based on boxplots were omitted from the data. The number of outliers was commonly restricted from 0 to 4. The distribution of the remaining data was tested for normality and homoscedasticity of residuals by Wilk-Shapiro and Levene’s tests, respectively. Since almost none of the data, even after log transformation, met the requirements for normality and homoscedasticity of residuals, the differences between groups were analyzed with the non-parametric Kruskal-Wallis and Mann-Whitney tests. Correlation between factors was tested with a Spearman’s rank order analysis. The numbers of abiotic soil factors were reduced by a principal component analysis.

## Results

### Abiotic Soil Factors

**Fig. 1:**
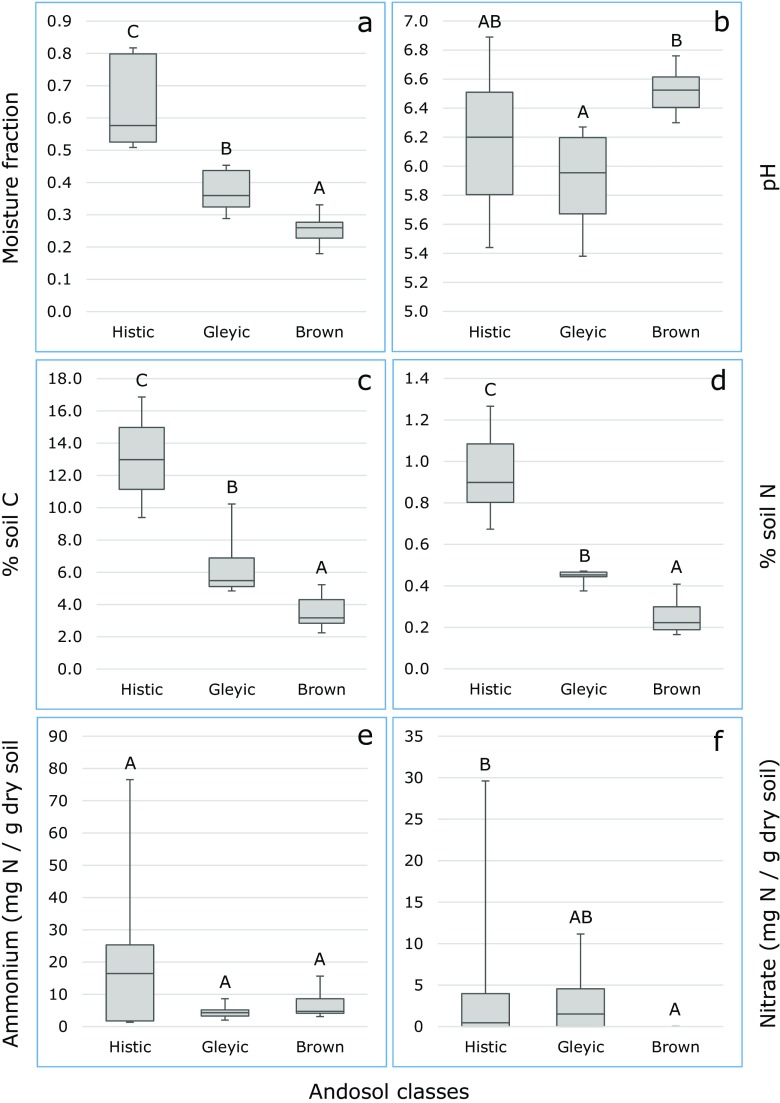
Boxplots of abiotic factors measured in Histic, Gleyic, or Brown Andosols: soil moisture fraction (**a**), soil pH-H_2_O (**b**), percentage of soil carbon (**c**), and nitrogen (**d**), KCl-extracted ammonium (**e**) and nitrate (**f**). Different *capitals above* the boxplots indicate significant differences (*p* < 0.035, Mann-Whitney test) between Andosol classes. Ammonium and nitrate are expressed as milligram N per gram dry soil

All abiotic soil factors measured were highly variable across and between sampling locations (Fig. 1). Based on carbon and moisture content, locations 1–3 were defined as Histic Andosols, locations 4 and 5 as Gleyic Andosols, and locations 6 and 7 as Brown Andosols (Supplementary Table S1). According to a non-parametric Kruskal-Wallis test, soil class had a significant effect on all measured abiotic soil factors, except on the amount of extracted ammonium. Moisture fraction, soil carbon percentage, and soil nitrogen percentage decreased significantly (*p* < 0.035) from the Histic to the Gleyic Andosols, and from the Gleyic to the Brown Andosols (Fig. 1a–d). Soil *C*/*N* ratio was significantly (*p* < 0.035) higher in Histic than in Gleyic Andosols, with Brown Andosols there between (not shown). Soil pH-H_2_O was significantly (*p* < 0.035) higher in Brown than in Gleyic Andosols, with Histic Andosols there between (Fig. 1b). The amount of nitrate was significantly (*p* < 0.035) lower in Brown than in Histic Andosols, with Gleyic Andosols there between (Fig. 1f). Nitrite was only detected in soil samples from the Histic Andosol locations 2 and 3 (2 and 1 sample, respectively). These locations contained also the highest amounts of ammonium (Fig. 1e).

According to a Spearman rank order correlation analysis, soil moisture content and the percentages of soil carbon and nitrogen were all significantly (*p* = 0.000) and positively correlated with each other (Supplementary Table S2). Soil pH-H_2_O was significantly (*p* = 0.000) and positively correlated with the *C*/*N* ratio in the soil, and significantly but negatively with the amounts of extracted ammonium (*p* = 0.005) and nitrite (*p* = 0.036). Nitrite was significantly and positively correlated with the amounts of extracted ammonium (*p* = 0.006) and with the percentages of soil carbon (*p* = 0.032) and nitrogen (*p* = 0.020). Finally, nitrate was significantly and positive correlated with the amount of extracted ammonium (*p* = 0.047).

### Copy Numbers of the *amoA* Genes

**Fig. 2:**
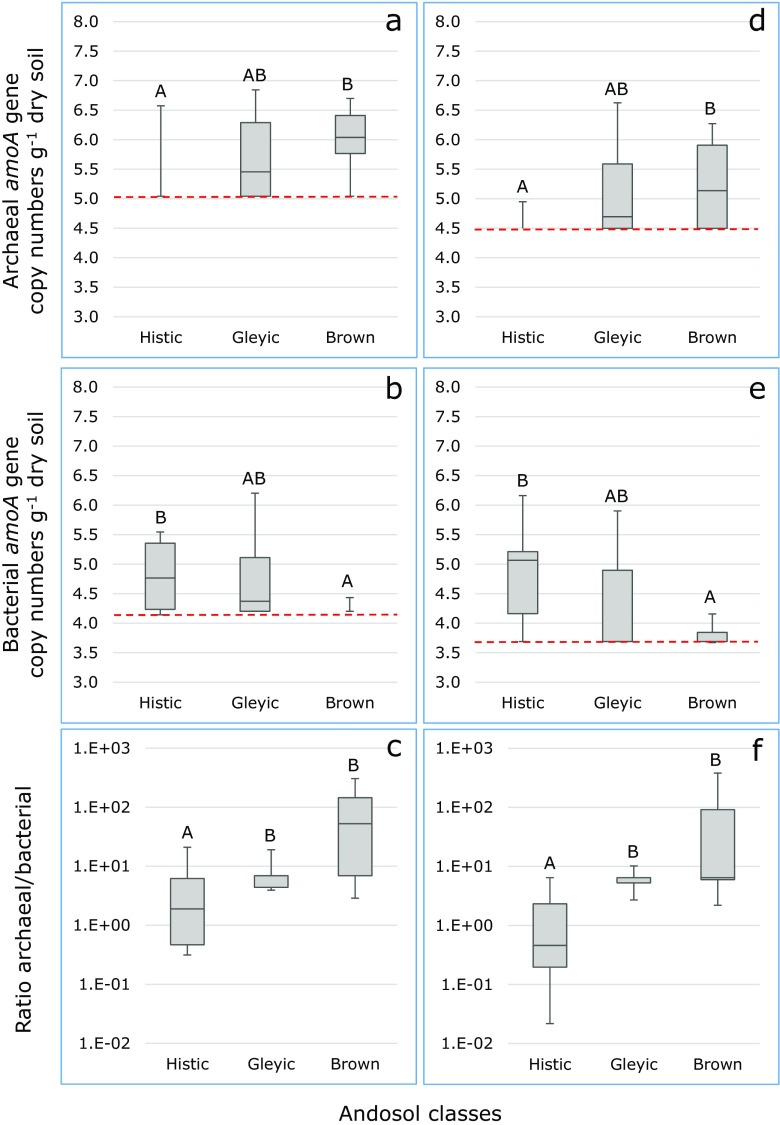
Boxplots of archaeal (**a**, **d**) and bacterial (**b**, **e**) *amoA* gene copy numbers and of their mutual ratios (**c**, **f**) determined with the old primer sets (**a**, **b**, **c**) or with the newly designed primer sets (**d**, **e**, **f**) in fresh soil samples collected from Histic, Gleyic, or Brown Andosols. Different capitals above the boxplots indicate significant differences (*p* < 0.035, Mann-Whitney test) between Andosol classes. *Red, broken lines* show the detection limits

Numbers of archaeal and bacterial *amoA* genes determined with either the old or the new primer set in fresh soil samples were significantly different for the Gleyic and Brown Andosols, but not for the Histic Andosols (Table 2). In Gleyic and Histic Andosols significantly higher numbers of archaeal and bacterial *amoA* were found with the old primer sets compared to the newly designed primer set. However, old or new primer set had no significant effect on the ratio between archaeal and bacterial *amoA* genes (Table 2). Irrespective of the primer sets applied, Andosol class had a significant effect on the abundance of the archaeal and bacterial *amoA* genes and on their mutual ratio. Brown Andosols contained significantly (*p* < 0.035) more archaeal *amoA* gene copies than Histic Andosols with Gleyic Andosols there between (Fig. 2a). Histic Andosols contained significantly (*p* < 0.035) higher bacterial *amoA* gene copies than Brown Andosols with again Gleyic Andosols there between (Fig. 2b). As a result, the AOA/AOB ratio was significantly (*p* < 0.035) higher in the Brown and Gleyic Andosols than in the Histic Andosols (Fig. 2c).

**Table 2:** Abundances of archaeal and bacterial *amoA* genes (copies per gram dry soil) determined with the old and new primer sets, respectively, in Icelandic andosols. Presented are the median values observed in each Andosol type

	Primer set	Andosol class		
		Histic	Gleyic	Brown
Archaeal amoA	Old	1.10E + 05	1.10E + 05	1.30E + 06
Archaeal amoA	New	3.20E + 04	3.20E + 04	2.50E + 05
*Significance between old and new primer sets*		*p = 0.147*	*p = 0.021*	*p = 0.000*
Bacterial amoA	Old	4.40E + 04	2.50E + 04	1.60E + 04
Bacterial amoA	New	1.10E + 05	6.10E + 03	4.90E + 03
*Significance between old and new primer sets*		*p = 0.150*	*p = 0.038*	*p = 0.000*
Archaeal versus bacterial amoA	Old	1.89	6.92	52.64
Archaeal versus bacterial amoA	New	0.46	6.45	6.45
*Significance old versus new*		*p = 0.054*	*p = 0.209*	*p = 0.401*

The newly designed primer sets yielded lower detection limits for both archaeal and bacterial *amoA* genes (Fig. 2). In 20% of the total soil samples, the old and new primer sets gave opposite outcomes for individual samples with respect to the presence or absence of *amoA* genes (Supplementary Table S3). Independent of the use of the old or the new primer sets, a relatively large number of samples did not show the presence of archaeal or bacterial *amoA* genes. The percentages of archaeal *amoA*-negative samples amounted to 51% of the soil samples when old and new primers were applied on fresh samples. The percentages of bacterial *amoA*-negative samples amounted to 34 and 37% of the fresh soil samples when the old and new primer sets were applied, respectively. Fresh samples from location 5 showed most *amoA*-negative samples (90%) and fresh samples from location 4 least (5%).

According to a Spearman rank order correlation analysis, the log of the abundances of archaeal *amoA* genes determined with the old or with the new primer set was significantly (*p* = 0.000) and positively correlated (Supplementary Table S2). This was also the case for the abundances of the bacterial *amoA* genes (*p* = 0.000). The correlation patterns obtained with old and new primer sets were similar with exception of the correlation of archaeal *amoA* genes and soil moisture content, and of bacterial *amoA* genes and soil pH-H_2_O (Supplementary Table S2).

The log of the archaeal *amoA* gene abundance was significantly but negatively correlated with the percentages of soil carbon (*p* = 0.000 for the old and 0.004 for the new primer set) and nitrogen (*p* = 0.001 for the old and 0.011 for the new primer set), and with the amounts of extracted nitrate (*p* = 0.014 for the old and 0.006 for the new primer set) (Supplementary Table S2). The log of the bacterial *amoA* gene abundance was significantly but positively correlated with soil moisture content (*p* = 0.014 for the old and 0.000 for the new primer set) and with the percentages of soil carbon (*p* = 0.037 for the old and 0.000 for the new primer set) and nitrogen (*p* = 0.021 for the old and 0.004 for the new primer set).

For checking the correctness of the primer sets applied with respect to the detection of *amoA* genes, gene fragments obtained with the original primer sets for the bacterial *amoA* gene were sequenced and all but one was mostly related to environmental clones of uncultured ammonia-oxidizing bacteria (Supplementary Table S4). The same results were obtained with the newly designed primer set for bacterial *amoA* gene. Gene fragments obtained with the new primer set for archaeal *amoA* gene showed sequences that were mostly related to environmental clones of uncultured ammonia-oxidizing archaea (Supplementary Table S5). The old primer set yielded the same results with respect of distribution of archaeal and bacterial *amoA* genes over the Andosol classes as the newly designed primer set.

### Potential Ammonia Oxidation Activities

**Fig. 3:**
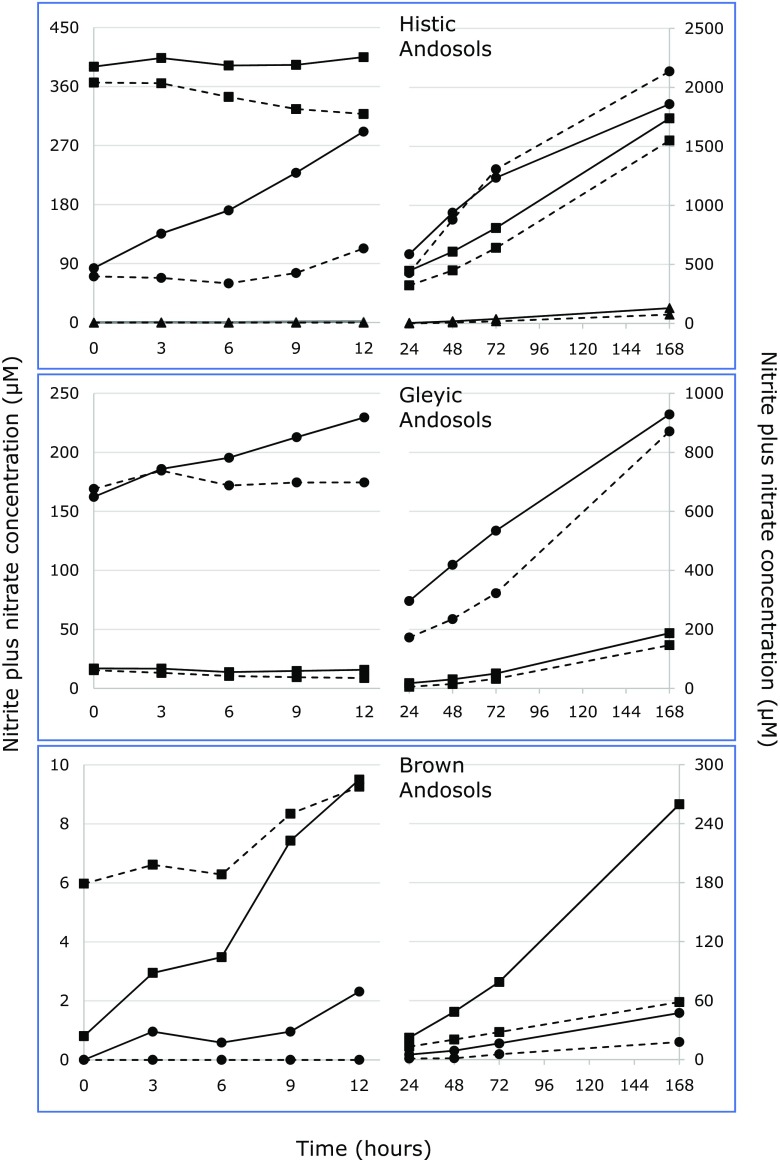
Changes in average (*n* = 5) nitrite plus nitrate concentrations in soil slurries from Histic Andosols (*upper panel*; *circles*: location 1, *squares*: location 2, *triangles*: location 3; *solid lines*: controls, *dashed lines*: plus ATU), from Gleyic Andosols (*middle panel*; *circles*: location 4, *squares*: location 5; *solid lines*: controls, *dashed lines*: plus ATU), and from Brown Andosols (*lower panel*; *circles*: location 6, *squares*: location 7; *solid lines*: controls, *dashed lines*: plus ATU). Slurries were incubated at 20 °C and 100 rpm in the presence of 1 mM ammonium sulfate. Average values and standard deviations are presented in Supplementary Table S6

Changes in nitrite plus nitrate (NO_x_) concentrations in slurries used to determine potential ammonia oxidation activities varied largely among the different sampling locations. This variation was independent on the Andosol class from which the samples were collected. In two of the seven locations tested, no potential ammonia oxidation activity was observed within the first 12 h of incubation at 20 °C. In the other soils, the activity was highly variable (Fig. 3). In the absence of the bacterial nitrification inhibitor ATU, the NO_x_ concentrations increased in slurries with samples from locations 1, 2, 4, and 7 during the first 12 h of incubation at 20 °C (see also Table 3). In slurries of the remaining locations, no net effect of incubation on the NO_x_ concentrations was observed during this time period. In the presence of ATU, the NO_x_ concentrations increased with samples from locations 1, 4 and 7, remained at the same level with samples from locations 3 and 6, or even decreased in slurries with samples from locations 2 and 5. Generally, the potential ammonia oxidation activities determined during the first 12 h in the presence of ATU were lower than those measured in the absence of ATU, but this was only significant for activities observed in slurries with samples from locations 1, 2, and 4 (Table 3).

**Table 3:** Median (*n* = 5) values of potential ammonia oxidation activities (nMol g^−1^ dry soil h^−1^) determined in Icelandic Andosols in the absence (control) or presence of the inhibitor allylthiourea (ATU). Different letters in one column indicate significant differences at the level *p* < 0.05

Location	Potential activities measured over the first 12 h of incubation			Potential activities measured from 24 to 72 h of incubation		
	Control	Plus ATU	Significance of the ATU effect	Control	Plus ATU	Significance of the ATU effect
1	39 c	6 bc	*p* = 0.027	121 bc	277 c	*p* = 0.465
2	8 a	−31 a	*p* = 0.014	85 c	87 bc	*p* = 0.754
3	0 a	0 bc	*p* = 0.136	3 a	3 a	*p* = 0.435
4	28 bc	4 bc	*p* = 0.025	43 b	37 b	*p* = 0.221
5	0 ab	−1 b	*p* = 0.119	4 a	5 a	*p* = 0.361
6	0 a	0 b	*p* = 0.136	1 a	0 a	*p* = 0.443
7	3 ab	1c	*p* = 0.176	9 b	2 a	*p* = 0.027

After 24 h of incubation of the soil slurries at 20 °C, NO_x_ concentrations generally increased (Fig. 3). ATU had a variable effect on the ammonia oxidation activities (Table 3). However, due to the large variation among the samples from one location, only samples from location 7 showed a significant and negative effect of ATU on the potential ammonia oxidation activity.

Irrespective of the absence or presence of the inhibitor allylthiourea, a Spearman rank order correlation analysis showed a significant and positive correlation between potential ammonia oxidation activities and soil moisture contents (*p* = 0.000), the percentages of soil carbon (*p* = 0.000) and nitrogen (*p* = 0.000), and the log of the bacterial *amoA* gene abundance (*p* = 0.000) (Supplementary Table S2).

## Discussion

### The Presence of Bacterial *amoA* Genes in Icelandic Andosols

The observation of bacterial *amoA* genes in freshly collected Icelandic Andosols rejects our hypothesis that these genes are below the detection limit of quantitative PCR analysis in these soils. One of the remarkable observations in the present study is the different distribution of archaeal and bacterial *amoA* genes over the Andosol classes. The dry, organic-poor Brown Andosols seem to favor the archaeal *amoA* genes, while the copy numbers of bacterial *amoA* genes were highest in the moist, organic-rich Histic Andosols. Hence, both types of *amoA* genes were clearly separated by the underlying soil characteristics. Of the environmental and edaphic factors that might stimulate archaeal over bacterial ammonia oxidizers [16, 30], pH and elevated temperature do not seem to play a role in the observed distribution of archaeal and bacterial *amoA* genes over the different Andosols. pH-H_2_O varied among the Andosol classes and the sampling locations had been selected remotely from any source of geothermal warming [8]. Although not statistically significant, the ammonium concentration tends to be higher in the Histic Andosols than in the other Andosols at the moment of sampling. Fluxes of mineralized ammonium may also happen during the year after an increase in temperature as shown for Histic Andosols [11]. If so, then bacterial ammonia oxidizers apparently profited most of it. Other factors they may select for archaeal ammonia oxidizers, i.e., oxygen deprivation and mixotrophy, prevailed most likely also in the moist, organic-rich Histic Andosols. In that case, the effect of increased ammonium availability apparently overruled potential oxygen limitation and mixotrophy in this Andosol class.

In the present study, we used two primer sets for establishing the presence of bacterial *amoA* genes in Andosols: The primer set that was also applied by our group before [8, 10] and a new primer set that had been designed to yield shorter gene fragments enabling a better judgment of the quality of the PCR products and therefore offering a better estimation of gene copy numbers. In general, the new primer sets for archaeal as well as bacterial *amoA* genes yielded not only lower detection limits but also lower absolute numbers. This phenomenon most probably relates to differences in the slope of the calibration curves. Notwithstanding the difference in absolute gene numbers, the significance of the differences between the Andosol classes was not affected by the choice of primer sets. With the primer sets for archaeal *amoA* genes, we observed this gene in 49% of the freshly collected soil samples when applying the old and new primers sets. When applying the old and new primer sets for bacterial *amoA* genes, we observed this gene in 44 and 66% of the total number of fresh soil samples, respectively. Notwithstanding the large number of *amoA*-negative samples of freshly collected soils, we are confident that bacterial *amoA* genes are present in Icelandic Histic Andosols. The abundances of archaeal and bacterial *amoA* genes are clearly at their detection limits in Icelandic Andosols. A comparable observation had been done by Alves and colleagues in Arctic soils [1].

The median number of 9.4 × 10^3^ archaeal *amoA* gene copies per gram dry soil observed by our group in the past in non-fertilized Histic Andosols of ambient temperature [8] was 2 orders of magnitude lower than the median value found for archaeal *amoA* gene copies with the old primer set in the present study and on average 3 orders of magnitude lower than found for this domain of ammonia oxidizers in Arctic soils [1, 4, 18, 24, 35]. Hence, numbers found by our group before were relatively low [8]. What could be the reason for these different observations with respect to the presence of bacterial *amoA* genes in the Icelandic Andosols in the present and past studies? In a former study, bacterial *amoA* genes came to the fore after some weeks of incubation in the presence of added ammonium [10]. Hence, they must have been present in the Histic Andosols studied then but were apparently under the detection limit of the qPCR method applied when fresh soil samples were analyzed. Taking into account an average archaeal to bacterial *amoA* gene copy number ratio of 30 as observed in our present study, the bacterial *amoA* genes in fresh Histic Andosols in our former study would have been 3.1 × 10^2^ and therefore likely below the detection limit. A reason for the large difference in archaeal *amoA* gene copy numbers between the former and the present study may be found in the application of different DNA extraction methods, which was manually in the past and mechanically with the Maxwell®16 DNA extractor in the present study.

Depending on the type of soil, i.e., soil with ambient temperature or geothermally warmed, fertilized or non-fertilized, the median pH values in our former study [8] varied from 7.22 to 8.67. The median pH values of the soils analyzed in the present study were more acidic and varied from 5.96 to 6.53. Since the cation exchange capacity in Andosols declines sharply with decreasing pH [44], ammonium might have been more available for ammonia-oxidizing microorganisms in the latter soil samples, although it remains doubtful if this would be sufficient for ammonia-oxidizing bacteria to compete successfully with the ammonia-oxidizing archaea that presumable will have a higher affinity for ammonia [30].

### Potential Ammonia Oxidation Activities

Potential ammonia oxidation activities were highly variable between the sampling locations. Irrespective of the time of measuring, i.e., during the first 12 h of incubation or during day 2 and day 3, the highest activities were observed in samples collected at locations 1, 2, and 4 belonging respectively to the Histic (locations 1 and 2) and the Gleyic Andosols (location 4). Low activities were encountered in samples from location 2 (Histic Andosol), location 5 (Gleyic Andosol), and location 6 (Brown Andosol). Hence, the potential ammonia oxidation activities do not seem to be affected by Andosol class primarily. Potential ammonia oxidation activities are significantly correlated with the abundance of the bacterial *amoA* gene, but not with the abundance of the archaeal *amoA* gene. From this correlation, it can be concluded that cells of bacterial ammonia oxidizers are more active in situ or more reactive towards freshly added ammonium than cells of archaeal ammonia oxidizers at the moment of sampling. It is well known that *amoA* gene copy numbers do not necessarily reflect in situ activities. In studies on the abundance and activity of ammonia oxidizers in microcosms filled with paddy soils and fertilized with ammonium and/or urea, for example, the ratios of archaeal *amoA* transcript-to-gene abundance was lower than those of bacterial *amoA* [19], and ratios between ^13^C-labeled archaeal to bacterial *amoA* gene copies representing the active cells were also lower than ratios between ^12^C-labeled archaeal to bacterial *amoA* gene copies representing the resting cells [46]. On the other hand, bacterial *amoA* gene abundance was significantly correlated with potential ammonia oxidation activities, whereas archaeal *amoA* gene abundance was not. This may imply that potential ammonia oxidation activities still follow the bacterial *amoA* gene copy numbers.

In line with the conclusion on ammonia-oxidizing bacteria being more reactive than ammonia-oxidizing archaea to the conditions present during the slurry measurements of potential ammonia oxidation activities, are the observed inhibition of potential activity during the first 12 h of incubation when the nitrification inhibitor allylthiourea (ATU) was added. ATU is assumed to inhibit specifically ammonia-oxidizing bacteria [25, 34, 38, 39], although it may also inhibit ammonia-oxidizing archaea partially [17, 25, 33, 34]. For samples from locations 1, 2, and 4 with the highest potential activities, the effect of ATU was significant. After this period of 12 h, the negative effect of ATU on potential ammonia oxidation became less visible. The reason for this is unclear. It is hard to imagine that ATU is degraded in the slurries within 1 day. However, inactivation of the inhibitor may also have been due to adsorption to soil particles [6]. The absence of an effect of ATU on potential ammonia oxidation in Icelandic Histic Andosols as reported before [10] may also have been caused by the relative long incubation period of weeks applied in that study. In their study on ammonia oxidation in mangrove soils, Wang and Gu [45] showed also that the effectiveness of ATU declined with incubation time: The inhibition of growth of ammonia-oxidizing bacteria lasted only for 5 days in the presence of 100 mg L^−1^ or more ATU. The authors suggested that ATU may easily be hydrolyzed in soil slurries. An alternative inhibitor for bacterial ammonia oxidation may be octyne, which presence in the headspace will prevent degradation by soil particles [39].

The potential ammonia oxidation activities determined in the present study at location 1 were below the potential ammonia oxidation activities of 1120 ± 440 nMol N g^−1^ dry soil h^−1^ measured by our group with samples from the same location in the past [10]. However, whereas the samples from our present study had been collected in May 2013, the samples from our former study had been collected in August 2010. In a ^15^N-tracing experiment with samples collected in this location, we observed a significant and positive effect of temperature on gross nitrogen nitrification rates [11]. Potential ammonia oxidation rates have seldom been determined in Sub-arctic grass- and heathland soils. Potential ammonia oxidation rates have more often been measured in Arctic soils and ranged from 0.16–9500 nMol N g^−1^ dry soil h^−1^ [1, 4, 18, 32, 47]. The large range in rates can at least partly be explained by the application of different methods, i.e., measuring the rate of accumulation of nitrite and nitrate (in the absence or presence of chlorate as inhibitor of nitrite oxidation) or determining the ^15^N-nitrate dilution rate, and by the use of different incubation temperatures. Nevertheless, the potential ammonia oxidation rates measured in the present study fall within the large range of Arctic rates.

### Concluding Remarks

Opposite to our hypothesis, bacterial *amoA* genes were detectable in Icelandic Andosols, although a high percentage of the soil samples remained negative with respect to the detection of these genes, but this also applied to the archaeal *amoA* genes, irrespective of the use of old or new primer sets. Hence, numbers of both microbial groups were apparently close to the detection limits of the old and newly designed primer sets that we applied. It cannot be excluded that other microorganisms, which are not covered by the primer sets used by us, are involved in the process of ammonia oxidation in Icelandic Andosols. Nitrite-oxidizing *Nitrospira* species that were recently recognized as ammonia-oxidizing bacteria [12, 20, 29] may be well adapted to the extreme low ammonium concentrations present in Icelandic Andosols [7, 13]. Finally, the DNA extraction method used by us may have been less appropriate for the physical nature of the Andosols, although the method applied in this study that used the Maxwell®16 DNA extractor may have been more suited than the manual method used in our previous publication [8–11].The numerical ratio between archaeal and bacterial *amoA* genes varied significantly between Andosol classes with relatively more bacterial *amoA* genes in the Histic Andosols and more archaeal *amoA* genes in the Brown Andosols. The median of the archaeal to bacterial *amoA* gene ratios were lower with the new primer sets than with the old primer sets, at least for the Histic and Gleyic Andosols. For the Histic Andosols, this may lead from an archaeal *amoA* gene-dominated system with the old primer sets towards a bacterial *amoA*-dominated system with the newly designed primer sets. However, the differences between both sets of primers were not significant (Table 2) and the mutual differences between the Andosols classes were independent on the primer sets used (Fig. 2). Nevertheless, absolute gene numbers depended on the primer sets used.

Both Andosol classes differ significantly from each other with respect to soil moisture content and soil organic matter. These soil parameters were higher in the Histic than in the Brown Andosols. Based on current knowledge, moist and organic-rich Histic Andosols with likely a more restricted oxygen supply may be more suitable for ammonia-oxidizing archaea than for ammonia-oxidizing bacteria to proliferate, which contradicts the observation of significantly less archaeal *amoA* genes in Histic Andosols. However, a relatively higher number of genes can be based not only on better growth performances but also on better survival in the absence of growth. Knowledge on a differential survival of cells with archaeal or bacterial *amoA* genes is largely an unexplored area. So, it may well be that ammonia-oxidizing bacteria survive better in the moist, organic-rich Histic Andosols, and ammonia-oxidizing archaea better in the dryer, organic-poor Brown Andosols.

In contrast to the abundances of archaeal and bacterial *amoA* genes that were significantly affected by the Andosols classes, the potential ammonia oxidation activities were not influenced by these classes. Nevertheless, potential ammonia oxidation activities were significantly and positively correlated with the abundances of the bacterial *amoA* genes at the different sampling locations. The activities were not significantly correlated with the archaeal *amoA* genes. The inhibition of potential ammonia oxidation activities by ATU, especially during the first 12 h of the slurry incubations, implies the dominance of bacteria in the process of ammonia oxidation in these slurries. If bacteria are also the dominant in situ ammonia-oxidizing microorganisms in Icelandic Andosols, ammonia-oxidizing archaea must make a living in a different way, for example, by the oxidation of organic nitrogen compounds as suggested already by others [27, 37].

Archaea are not entirely excluded from ammonia oxidation in Icelandic Andosols as we have shown in the past by applying ^13^C–CO_2_ stable isotope-labeling in microcosms filled with Histic Andosols [9]. At low and high backgrounds of ammonium, archaeal *amoA* genes got labeled after 14 and 28 days. However, a possible role of bacteria in ammonia oxidation remained hidden in that study due to the inability to detect bacterial *amoA* genes in that time.

## Electronic supplementary material

Supplementary Table S1 (DOCX 12 kb)

Supplementary Table S2 (DOCX 16 kb)

Supplementary Table S3 (DOCX 13 kb)

Supplementary Table S4 (DOCX 17 kb)

Supplementary Table S5 (DOCX 17 kb)

Supplementary Table S6 (DOCX 18 kb)
