# Supplementary material for: Numerical Relationships Between Archaeal and Bacterial amoA Genes Vary by Icelandic Andosol Classes
Source: Microb Ecol. 2017 Jul 13;75(1):204–15. doi: 10.1007/s00248-017-1032-9 (PMC5742608; doi:10.1007/s00248-017-1032-9)
Supplement: Supplementary file 1 — (DOCX 12 kb) [file 248_2017_1032_MOESM1_ESM.docx]

Supplementary Table S1: Geographical, vegetation, soil and geological characteristics of the sampling locations in southwestern Iceland.

| Location | Geographical place | Coordinates | Vegetation | Soil classification | Geological epoch | Age |
| --- | --- | --- | --- | --- | --- | --- |
| 1 | Graendalur valley | +64.024385,  -21.209344 | sedges | Histic Andosol | Upper Pleistocene | < 0.8 M year |
| 2 | South of Selfors | +63.892373,  -21.0768655 | grasses | Histic Andosol | Postglacial prehistoric | > AD 871 |
| 3 | South of Hveragerdi | +63.950134,  -21.160658 | grasses | Histic Andosol | Upper Pliocene and Lower Pleistocene | 0.8 – 3.3 M year |
| 4 | Graendalur valley | +64.024385,  -21.209344 | grasses | Gleyic Andosol | Upper Pleistocene | < 0.8 M year |
| 5 | South of Hella | +63.800681,  -20.437714 | grasses with mosses | Gleyic Andosol |  | < AD 871 |
| 6 | Skardsheidi peninsula | +64.316148,  -21.985722 | grasses with mosses | Brown Andosol | Upper Tertiary | > 3.3 M year |
| 7 | Kjos peninsula | +64.331432,  -21.518454 | Heather with mosses | Brown Andosol | Upper Pliocene and Lower Pleistocene | 0.8 – 3.3 M year |
