# Supplementary material for: Numerical Relationships Between Archaeal and Bacterial amoA Genes Vary by Icelandic Andosol Classes
Source: Microb Ecol. 2017 Jul 13;75(1):204–15. doi: 10.1007/s00248-017-1032-9 (PMC5742608; doi:10.1007/s00248-017-1032-9)
Supplement: Supplementary file 2 — (DOCX 16 kb) [file 248_2017_1032_MOESM2_ESM.docx]

Supplementary Table S2: Spearman Rank Order correlations between abiotic and biotic soil characteristics. Spearman rho’s are shown above the diagonal, the corresponding p values below the diagonal with significant p values in red.

|  | Moisture content | pH-H2O | Total soil carbon | Total soil nitrogen | Extracted ammonium | Extracted nitrate | LogAOA old primer set | LogAOA new primer set | LogAOB old primer set | LogAOB new primer set | AOA/AOB old primer set | AOA/AOB new primer set | PAA minus ATU | PAA plus ATU |
| --- | --- | --- | --- | --- | --- | --- | --- | --- | --- | --- | --- | --- | --- | --- |
| Moisture content |  | -0.305 | 0.942 | 0.953 | 0.027 | 0.229 | -0.434 | -0.312 | 0.442 | 0.655 | -0.613 | -0.829 | 0.667 | 0.722 |
| pH-H2O | 0.096 |  | -0.231 | -0.276 | -0.468 | -0.207 | -0.075 | -0.211 | -0.537 | -0.212 | 0.399 | -0.033 | -0.226 | -0.219 |
| Total soil carbon | 0.000 | 0.189 |  | 0.993 | 0.061 | 0.272 | -0.619 | -0.502 | 0.365 | 0.593 | -0.651 | -0.827 | 0.606 | 0.682 |
| Total soil nitrogen | 0.000 | 0.133 | 0.000 |  | 0.101 | 0.328 | -0.595 | -0.473 | 0.421 | 0.627 | -0.673 | -0.842 | 0.628 | 0.694 |
| Extracted ammonium | 0.888 | 0.005 | 0.737 | 0.595 |  | 0.315 | -0.244 | 0.038 | 0.337 | -0.008 | -0.418 | 0.070 | -0.090 | -0.178 |
| Extracted nitrate | 0.232 | 0.247 | 0.133 | 0.082 | 0.079 |  | -0.445 | -0.487 | -0.242 | -0.098 | -0.131 | -0.086 | -0.293 | -0.202 |
| LogAOA old primer set | 0.021 | 0.683 | 0.000 | 0.001 | 0.186 | 0.014 |  | 0.714 | -0.031 | -0.254 | 0.595 | 0.520 | -0.056 | -0.104 |
| LogAOA new primer set | 0.107 | 0.246 | 0.004 | 0.011 | 0.838 | 0.006 | 0.000 |  | 0.282 | 0.009 | 0.168 | 0.393 | 0.106 | 0.060 |
| LogAOB old primer set | 0.014 | 0.001 | 0.037 | 0.021 | 0.055 | 0.183 | 0.870 | 0.125 |  | 0.742 | -0.740 | -0.396 | 0.674 | 0.590 |
| LogAOB new primer set | 0.000 | 0.237 | 0.000 | 0.000 | 0.965 | 0.599 | 0.176 | 0.962 | 0.000 |  | -0.707 | -0.782 | 0.779 | 0.765 |
| AOA/AOB old primer set | 0.000 | 0.022 | 0.000 | 0.000 | 0.017 | 0.484 | 0.000 | 0.367 | 0.000 | 0.000 |  | 0.626 | -0.497 | -0.521 |
| AOA/AOB new primer set | 0.000 | 0.861 | 0.000 | 0.000 | 0.717 | 0.662 | 0.004 | 0.032 | 0.034 | 0.000 | 0.000 |  | -0.688 | -0.663 |
| PAA minus ATU | 0.000 | 0.205 | 0.000 | 0.000 | 0.622 | 0.109 | 0.768 | 0.578 | 0.000 | 0.000 | 0.004 | 0.000 |  | 0.964 |
| PAA plus ATU | 0.000 | 0.207 | 0.000 | 0.000 | 0.315 | 0.259 | 0.571 | 0.743 | 0.000 | 0.000 | 0.002 | 0.000 | 0.000 |  |

^1^ p.s. = primer set; ^2^ PAA plus ATU *versus* PAA minus ATU
