# Supplementary material for: Numerical Relationships Between Archaeal and Bacterial amoA Genes Vary by Icelandic Andosol Classes
Source: Microb Ecol. 2017 Jul 13;75(1):204–15. doi: 10.1007/s00248-017-1032-9 (PMC5742608; doi:10.1007/s00248-017-1032-9)
Supplement: Supplementary file 3 — (DOCX 13 kb) [file 248_2017_1032_MOESM3_ESM.docx]

Supplementary Table S3: Numbers of archaeal and bacterial amoA genes detected in fresh soil samples from Icelandic Andosols

| Sample number | Location | Archaeal *amoA* | | Bacterial *amoA* | |
| --- | --- | --- | --- | --- | --- |
|  |  | old primer set | new primer set | old primer set | new primer set |
| 1A | 1 | 3.75E+06 | 1.32E+05 | negative | negative |
| 1B | 1 | 3.34E+05 | negative | negative | 2.11E+04 |
| 1C | 1 | negative | negative | 1.77E+04 | 5.16E+05 |
| 1D | 1 | negative | negative | 5.83E+04 | 5.19E+05 |
| 1E | 1 | negative | negative | 8.00E+04 | 1.45E+06 |
| 2A | 2 | negative | negative | 3.21E+05 | 1.06E+05 |
| 2B | 2 | negative | negative | 2.36E+05 | 1.67E+05 |
| 2C | 2 | negative | 4.81E+04 | 2.66E+05 | 1.58E+05 |
| 2D | 2 | negative | negative | 3.51E+05 | 1.43E+05 |
| 2E | 2 | 1.26E+07 | 3.98E+05 | 2.18E+05 | 1.59E+05 |
| 3A | 3 | negative | 8.87E+04 | 2.45E+04 | 5.01E+04 |
| 3B | 3 | negative | negative | 1.66E+04 | negative |
| 3C | 3 | negative | negative | 3.38E+04 | 1.00E+04 |
| 3D | 3 | 1.36E+05 | negative | 1.39E+04 | negative |
| 3E | 3 | negative | 7.14E+04 | 2.18E+05 | 1.16E+05 |
| 4A | 4 | 5.19E+06 | 4.04E+05 | 2.36E+04 | negative |
| 4B | 4 | 2.45E+06 | 7.81E+04 | 1.29E+05 | 7.65E+03 |
| 4C | 4 | 9.78E+05 | 8.05E+05 | 2.49E+05 | 1.12E+05 |
| 4D | 4 | 7.35E+05 | 3.44E+05 | 3.86E+04 | 7.89E+04 |
| 4E | 4 | 6.98E+06 | 4.21E+06 | 1.59E+06 | 7.98E+05 |
| 5A | 5 | negative | negative | negative | negative |
| 5B | 5 | negative | negative | negative | negative |
| 5C | 5 | negative | negative | negative | negative |
| 5D | 5 | negative | negative | 2.52E+04 | 1.16E+04 |
| 5E | 5 | negative | negative | negative | negative |
| 6A | 6 | 5.81E+05 | 3.63E+04 | negative | 7.95E+03 |
| 6B | 6 | negative | negative | negative | negative |
| 6C | 6 | 4.86E+06 | 1.05E+05 | negative | 4.69E+03 |
| 6D | 6 | 2.57E+06 | negative | negative | negative |
| 6E | 6 | negative | negative | negative | 1.44E+04 |
| 7A | 7 | 4.99E+06 | 4.48E+05 | 2.72E+04 | negative |
| 7B | 7 | 1.09E+06 | 1.87E+06 | negative | negative |
| 7C | 7 | 8.56E+05 | 8.06E+05 | 2.98E+05 | 1.35E+05 |
| 7D | 7 | 1.53E+06 | 1.16E+06 | negative | 7.01E+03 |
| 7E | 7 | negative | 1.37E+05 | 2.01E+04 | negative |
| Detection limit |  | 1.10E+05 | 3.15E+04 | 1.59E+04 | 4.89E+03 |
