# Supplementary material for: Numerical Relationships Between Archaeal and Bacterial amoA Genes Vary by Icelandic Andosol Classes
Source: Microb Ecol. 2017 Jul 13;75(1):204–15. doi: 10.1007/s00248-017-1032-9 (PMC5742608; doi:10.1007/s00248-017-1032-9)
Supplement: Supplementary file 4 — (DOCX 17 kb) [file 248_2017_1032_MOESM4_ESM.docx]

Supplementary Table S4: Environmental clones of the bacterial *amoA* gene, which are most close to sequences obtained from Icelandic Andosols.

| Sampling location | Primer set | Clone direction | Sequence ID | Description closest relative | Identical | Accession | Clone origin | Country |
| --- | --- | --- | --- | --- | --- | --- | --- | --- |
| 4 | Old | M13for | 1666BAB001-11 | Uncultured bacterium clone DH03fR_09 | 98% | [HM131552.1](http://www.ncbi.nlm.nih.gov/nucleotide/299480872?report=genbank&log$=nucltop&blast_rank=1&RID=PP04C59N015) | Duke forest soil | USA |
| 4 | Old | M13for | 1666BAB001-12 | Uncultured bacterium clone NB-CT-82 | 98% | [KC010732.1](http://www.ncbi.nlm.nih.gov/nucleotide/442797526?report=genbank&log$=nucltop&blast_rank=1&RID=PP04C59N015) | Grassland soil | New Zealand |
| 4 | Old | M13for | 1666BAB001-13 | Uncultured bacterium clone NB-CT-82 | 98% | [KC010732.1](http://www.ncbi.nlm.nih.gov/nucleotide/442797526?report=genbank&log$=nucltop&blast_rank=1&RID=PP04C59N015) | Grassland soil | New Zealand |
| 4 | Old | M13for | 1666BAB001-14 | Uncultured bacterium clone NB-CT-82 | 98% | [KC010732.1](http://www.ncbi.nlm.nih.gov/nucleotide/442797526?report=genbank&log$=nucltop&blast_rank=1&RID=PP04C59N015) | Grassland soil | New Zealand |
| 4 | Old | M13for | 1666BAB001-15 | Uncultured bacterium clone DH03fR_09 | 99% | [HM131552.1](http://www.ncbi.nlm.nih.gov/nucleotide/299480872?report=genbank&log$=nucltop&blast_rank=1&RID=PP39FM60015) | Duke forest soil | USA |
| 2 | Old | M13for | 1666BAB001-16 | No significant similarity found. |  |  |  |  |
| 2 | Old | M13for | 1666BAB001-17 | Uncultured bacterium clone DH03fR_09 | 98% | [HM131552.1](http://www.ncbi.nlm.nih.gov/nucleotide/299480872?report=genbank&log$=nucltop&blast_rank=1&RID=PS3DMA52015) | Duke forest soil | USA |
| 2 | Old | M13for | 1666BAB001-18 | Uncultured ammonia-oxidizing bacterium clone AOBu-A1C2 | 98% | [GQ143471.1](http://www.ncbi.nlm.nih.gov/nucleotide/256486839?report=genbank&log$=nucltop&blast_rank=1&RID=PS3DMA52015) | Soil | China |
| 2 | Old | M13for | 1666BAB001-19 | Uncultured bacterium clone DH03fR_09 | 98% | [HM131552.1](http://www.ncbi.nlm.nih.gov/nucleotide/299480872?report=genbank&log$=nucltop&blast_rank=1&RID=PS3DMA52015) | Duke forest soil | USA |
| 2 | Old | M13for | 1666BAB001-20 | Uncultured bacterium clone UT-CLY_02 | 99% | [DQ480781.1](http://www.ncbi.nlm.nih.gov/nucleotide/94470534?report=genbank&log$=nucltop&blast_rank=1&RID=PS3DMA52015) | Soil | USA |
| 2 | Old | M13rev | 1666BAB001-36 | Uncultured bacterium clone DH03fR_09 | 98% | [HM131552.1](http://www.ncbi.nlm.nih.gov/nucleotide/299480872?report=genbank&log$=nucltop&blast_rank=1&RID=PS3DMA52015) | Duke forest soil | USA |
| 2 | Old | M13rev | 1666BAB001-37 | Uncultured bacterium clone DH03fR_09 | 98% | [HM131552.1](http://www.ncbi.nlm.nih.gov/nucleotide/299480872?report=genbank&log$=nucltop&blast_rank=1&RID=PS3DMA52015) | Duke forest soil | USA |
| 2 | Old | M13rev | 1666BAB001-38 | Uncultured ammonia-oxidizing bacterium clone AOBu-A1C2 | 98% | [GQ143471.1](http://www.ncbi.nlm.nih.gov/nucleotide/256486839?report=genbank&log$=nucltop&blast_rank=1&RID=PS3DMA52015) | Soil | China |
| 2 | Old | M13rev | 1666BAB001-39 | Uncultured bacterium clone DH03fR_09 | 98% | [HM131552.1](http://www.ncbi.nlm.nih.gov/nucleotide/299480872?report=genbank&log$=nucltop&blast_rank=1&RID=PS3DMA52015) | Duke forest soil | USA |
| 2 | Old | M13rev | 1666BAB001-40 | Uncultured bacterium clone UT-CLY_02 | 99% | [DQ480781.1](http://www.ncbi.nlm.nih.gov/nucleotide/94470534?report=genbank&log$=nucltop&blast_rank=1&RID=PS3DMA52015) | Soil | USA |
| 4 | Old | M13rev | 1666BAB001-31 | Uncultured bacterium clone DH03fR_09 | 98% | [HM131552.1](http://www.ncbi.nlm.nih.gov/nucleotide/299480872?report=genbank&log$=nucltop&blast_rank=1&RID=PS3DMA52015) | Duke forest soil | USA |
| 4 | Old | M13rev | 1666BAB001-32 | Uncultured bacterium clone NB-CT-82 | 98% | [KC010732.1](http://www.ncbi.nlm.nih.gov/nucleotide/442797526?report=genbank&log$=nucltop&blast_rank=1&RID=PS3DMA52015) | Grassland soil | New Zealand |
| 4 | Old | M13rev | 1666BAB001-33 | Uncultured bacterium clone NB-CT-82 | 98% | [KC010732.1](http://www.ncbi.nlm.nih.gov/nucleotide/442797526?report=genbank&log$=nucltop&blast_rank=1&RID=PS3DMA52015) | Grassland soil | New Zealand |
| 4 | Old | M13rev | 1666BAB001-34 | Uncultured bacterium clone NB-CT-82 | 99% | [KC010732.1](http://www.ncbi.nlm.nih.gov/nucleotide/442797526?report=genbank&log$=nucltop&blast_rank=1&RID=PS3DMA52015) | Grassland soil | New Zealand |
| 4 | Old | M13rev | 1666BAB001-35 | Uncultured bacterium clone DH03fR_09 | 99% | [HM131552.1](http://www.ncbi.nlm.nih.gov/nucleotide/299480872?report=genbank&log$=nucltop&blast_rank=1&RID=PS3DMA52015) | Duke forest soil | USA |
| 1 | New | M13for | 1666BAB001-21 | Uncultured bacterium clone AOB-D2- | 97% | [KP197441.1](http://www.ncbi.nlm.nih.gov/nucleotide/800923027?report=genbank&log$=nucltop&blast_rank=1&RID=PS3DMA52015) | Soil of Erhai lake basin | China |
| 1 | New | M13for | 1666BAB001-22 | Uncultured bacterium clone AOB-D2-9 | 96% | [KP197441.1](http://www.ncbi.nlm.nih.gov/nucleotide/800923027?report=genbank&log$=nucltop&blast_rank=1&RID=PS3DMA52015) | Soil of Erhai lake basin | China |
| 1 | New | M13for | 1666BAB001-23 | Uncultured bacterium clone AOB-D2-9 | 96% | [KP197441.1](http://www.ncbi.nlm.nih.gov/nucleotide/800923027?report=genbank&log$=nucltop&blast_rank=1&RID=PS3DMA52015) | Soil of Erhai lake basin | China |
| 1 | New | M13for | 1666BAB001-24 | Uncultured bacterium clone LT-600_43 | 97% | [DQ480853.1](http://www.ncbi.nlm.nih.gov/nucleotide/94470678?report=genbank&log$=nucltop&blast_rank=1&RID=PS3DMA52015) | Soil | USA |
| 1 | New | M13for | 1666BAB001-25 | Uncultured bacterium clone LT-600_43 | 97% | [DQ480853.1](http://www.ncbi.nlm.nih.gov/nucleotide/94470678?report=genbank&log$=nucltop&blast_rank=1&RID=PS3DMA52015) | Soil | USA |
| 7 | New | M13for | 1666BAB001-26 | Uncultured bacterium clone JH41 | 99% | [KJ144239.1](http://www.ncbi.nlm.nih.gov/nucleotide/590124084?report=genbank&log$=nucltop&blast_rank=1&RID=PS3DMA52015) | Lake sediments | China |
| 7 | New | M13for | 1666BAB001-27 | Uncultured bacterium clone TC3 | 98% | [KJ160450.1](http://www.ncbi.nlm.nih.gov/nucleotide/597589933?report=genbank&log$=nucltop&blast_rank=1&RID=PS3DMA52015) | Lake sediments | China |
| 7 | New | M13for | 1666BAB001-28 | Uncultured bacterium clone K43_74 | 99% | [FN600122.1](http://www.ncbi.nlm.nih.gov/nucleotide/270286469?report=genbank&log$=nucltop&blast_rank=1&RID=PS3DMA52015) | Rice field | Italy |
| 7 | New | M13for | 1666BAB001-29 | No significant similarity found |  |  |  |  |
| 7 | New | M13for | 1666BAB001-30 | Uncultured ammonia oxidising bacterium clone JX_AOB_6-5 | 98% | [HQ594968.1](http://www.ncbi.nlm.nih.gov/nucleotide/333973427?report=genbank&log$=nucltop&blast_rank=2&RID=PS3DMA52015) | Paddy field soil | China |
| 7 | New | M13for | 1666BAB001-48 | Uncultured bacterium clone Late_DF_62_56_U05_C01_43 | 96% | [FJ529733.1](http://www.ncbi.nlm.nih.gov/nucleotide/222146132?report=genbank&log$=nucltop&blast_rank=1&RID=PG7XC8K9015) | Soil | USA |
