# Supplementary material for: Numerical Relationships Between Archaeal and Bacterial amoA Genes Vary by Icelandic Andosol Classes
Source: Microb Ecol. 2017 Jul 13;75(1):204–15. doi: 10.1007/s00248-017-1032-9 (PMC5742608; doi:10.1007/s00248-017-1032-9)
Supplement: Supplementary file 5 — (DOCX 17 kb) [file 248_2017_1032_MOESM5_ESM.docx]

Supplementary Table S5: Environmental clones of the archaeal *amoA* gene, which are most close to sequences obtained from Icelandic Andosols

| Sampling location | Primer set | Clone direction | Sequence ID | Description closest relative | Identical | Accession | Clone origin | Country |
| --- | --- | --- | --- | --- | --- | --- | --- | --- |
| 4 | New | M13for | 1666BAB001-1 | Uncultured archaeon clone 18C_87 | 98% | [JQ403851.1](http://www.ncbi.nlm.nih.gov/nucleotide/375341577?report=genbank&log$=nucltop&blast_rank=1&RID=PG7XC8K9015) | Grassland soil | Iceland |
| 4 | New | M13for | 1666BAB001-2 | Uncultured archaeon clone DX16-47 | 98% | [KF709842.1](http://www.ncbi.nlm.nih.gov/nucleotide/694190487?report=genbank&log$=nucltop&blast_rank=1&RID=PG7XC8K9015) | Alpine soil | China |
| 4 | New | M13for | 1666BAB001-3 | Uncultured archaeon clone Carex-aoa-15 | 98% | [KF754178.1](http://www.ncbi.nlm.nih.gov/nucleotide/575797016?report=genbank&log$=nucltop&blast_rank=1&RID=PG7XC8K9015) | Alpine meadow soil | China |
| 4 | New | M13for | 1666BAB001-4 | Uncultured archaeon clone 3-29 | 97% | [KF003989.1](http://www.ncbi.nlm.nih.gov/nucleotide/514230021?report=genbank&log$=nucltop&blast_rank=1&RID=PG7XC8K9015) | Alpine meadow soil | China |
| 4 | New | M13for | 1666BAB001-5 | Uncultured archaeon clone 25#48 | 97% | [HM122236.1](http://www.ncbi.nlm.nih.gov/nucleotide/302121748?report=genbank&log$=nucltop&blast_rank=1&RID=PG7XC8K9015) | Soil | China |
| 7 | New | M13for | 1666BAB001-6 | Uncultured archaeon clone 18C_87 | 97% | [JQ403851.1](http://www.ncbi.nlm.nih.gov/nucleotide/375341577?report=genbank&log$=nucltop&blast_rank=1&RID=PG7XC8K9015) | Grassland soil | Iceland |
| 7 | New | M13for | 1666BAB001-7 | Uncultured archaeon clone DX16-47 | 97% | [KF709842.1](http://www.ncbi.nlm.nih.gov/nucleotide/694190487?report=genbank&log$=nucltop&blast_rank=1&RID=PG7XC8K9015) | Alpine soil | China |
| 7 | New | M13for | 1666BAB001-8 | Uncultured archaeon clone DX16-47 | 97% | [KF709842.1](http://www.ncbi.nlm.nih.gov/nucleotide/694190487?report=genbank&log$=nucltop&blast_rank=1&RID=PG7XC8K9015) | Alpine soil | China |
| 7 | New | M13for | 1666BAB001-9 | Uncultured archaeon clone 3-29 | 98% | [KF003989.1](http://www.ncbi.nlm.nih.gov/nucleotide/514230021?report=genbank&log$=nucltop&blast_rank=1&RID=PG7XC8K9015) | Alpine meadow soil | China |
| 7 | New | M13for | 1666BAB001-10 | Uncultured ammonia-oxidizing archaeon clone AOAd-743 | 98% | [GQ142511.1](http://www.ncbi.nlm.nih.gov/nucleotide/256483879?report=genbank&log$=nucltop&blast_rank=1&RID=PG7XC8K9015) | Soil | China |
| 4 | New | M13rev | 1666BAB001-41 | Uncultured archaeon clone 25#48 | 96% | [HM122236.1](http://www.ncbi.nlm.nih.gov/nucleotide/302121748?report=genbank&log$=nucltop&blast_rank=1&RID=PG7XC8K9015) | Soil | China |
| 4 | New | M13rev | 1666BAB001-42 | Uncultured archaeon clone Carex-aoa-15 | 96% | [KF754178.1](http://www.ncbi.nlm.nih.gov/nucleotide/575797016?report=genbank&log$=nucltop&blast_rank=1&RID=PG7XC8K9015) | Alpine meadow soil | China |
| 4 | New | M13rev | 1666BAB001-43 | Uncultured archaeon clone Carex-aoa-15 | 98% | [KF754178.1](http://www.ncbi.nlm.nih.gov/nucleotide/575797016?report=genbank&log$=nucltop&blast_rank=1&RID=PG7XC8K9015) | Alpine meadow soil | China |
| 4 | New | M13rev | 1666BAB001-44 | Uncultured crenarchaeote clone XH-M-Seq2 | 97% | [JQ698589.1](http://www.ncbi.nlm.nih.gov/nucleotide/387913376?report=genbank&log$=nucltop&blast_rank=1&RID=PG7XC8K9015) | Sediment | China |
| 7 | New | M13rev | 1666BAB001-45 | Uncultured archaeon clone DX16-47 | 97% | [KF709842.1](http://www.ncbi.nlm.nih.gov/nucleotide/694190487?report=genbank&log$=nucltop&blast_rank=1&RID=PG7XC8K9015) | Alpine soil | China |
| 7 | New | M13rev | 1666BAB001-46 | Uncultured archaeon clone DX16-45 | 98% | [KF709840.1](http://www.ncbi.nlm.nih.gov/nucleotide/694190483?report=genbank&log$=nucltop&blast_rank=1&RID=PG7XC8K9015) | Alpine soil | China |
| 7 | New | M13rev | 1666BAB001-47 | Uncultured archaeon clone DX16-47 | 97% | [KF709842.1](http://www.ncbi.nlm.nih.gov/nucleotide/694190487?report=genbank&log$=nucltop&blast_rank=1&RID=PG7XC8K9015) | Alpine soil | China |
