# Supplementary material for: Numerical Relationships Between Archaeal and Bacterial amoA Genes Vary by Icelandic Andosol Classes
Source: Microb Ecol. 2017 Jul 13;75(1):204–15. doi: 10.1007/s00248-017-1032-9 (PMC5742608; doi:10.1007/s00248-017-1032-9)
Supplement: Supplementary file 6 — (DOCX 18 kb) [file 248_2017_1032_MOESM6_ESM.docx]

Supplementary Table S6: Average values (AVG, n=5), standard deviations (SD) and coefficients of variation (CV) belonging to the measurements of potential nitrification activities in Icelandic Andosols.

| Location | Andosol class | ATU addition |  | Incubation time (hours) | | | | | | | | |
| --- | --- | --- | --- | --- | --- | --- | --- | --- | --- | --- | --- | --- |
|  |  |  |  | 0 | 3 | 6 | 9 | 12 | 24 | 48 | 72 | 168 |
| 1 | Histic | - | AVG | 83 | 136 | 171 | 228 | 291 | 586 | 938 | 1234 | 1859 |
|  |  |  | SD | 75 | 140 | 186 | 249 | 319 | 636 | 814 | 902 | 841 |
|  |  |  | CV (%) | 90 | 104 | 109 | 109 | 109 | 108 | 87 | 73 | 45 |
|  |  | + | AVG | 70 | 68 | 60 | 76 | 113 | 427 | 879 | 1307 | 2136 |
|  |  |  | SD | 104 | 110 | 102 | 123 | 173 | 571 | 762 | 934 | 949 |
|  |  |  | CV (%) | 147 | 161 | 170 | 162 | 153 | 134 | 87 | 71 | 44 |
| 2 | Histic | - | AVG | 390 | 404 | 392 | 393 | 405 | 445 | 608 | 809 | 1737 |
|  |  |  | SD | 161 | 166 | 168 | 158 | 164 | 174 | 203 | 213 | 388 |
|  |  |  | CV (%) | 41 | 41 | 43 | 40 | 41 | 39 | 33 | 26 | 22 |
|  |  | + | AVG | 366 | 365 | 344 | 326 | 318 | 322 | 449 | 640 | 1550 |
|  |  |  | SD | 171 | 173 | 169 | 161 | 158 | 123 | 90 | 80 | 237 |
|  |  |  | CV (%) | 47 | 47 | 49 | 50 | 50 | 38 | 20 | 12 | 15 |
| 3 | Histic | - | AVG | 0 | 0 | 0 | 1 | 1 | 5 | 19 | 38 | 130 |
|  |  |  | SD | 0 | 1 | 0 | 1 | 2 | 6 | 23 | 45 | 152 |
|  |  |  | CV (%) | n.a.^1^ | 172 | 125 | 130 | 123 | 124 | 122 | 120 | 117 |
|  |  | + | AVG | 0 | 0 | 0 | 0 | 0 | 0 | 8 | 19 | 75 |
|  |  |  | SD | 0 | 0 | 0 | 0 | 0 | 1 | 10 | 23 | 84 |
|  |  |  | CV (%) | n.a. | n.a. | n.a. | n.a. | n.a. | 176 | 124 | 124 | 112 |
| 4 | Gleyic | - | AVG | 162 | 186 | 195 | 213 | 230 | 296 | 419 | 534 | 929 |
|  |  |  | SD | 67 | 75 | 73 | 78 | 84 | 94 | 110 | 125 | 175 |
|  |  |  | CV (%) | 41 | 40 | 38 | 37 | 37 | 32 | 26 | 23 | 19 |
|  |  | + | AVG | 169 | 185 | 172 | 175 | 175 | 172 | 235 | 323 | 871 |
|  |  |  | SD | 67 | 75 | 70 | 68 | 73 | 65 | 75 | 87 | 236 |
|  |  |  | CV (%) | 39 | 41 | 41 | 39 | 42 | 38 | 32 | 27 | 27 |
| 5 | Gleyic | - | AVG | 17 | 17 | 14 | 15 | 16 | 18 | 31 | 51 | 187 |
|  |  |  | SD | 27 | 25 | 22 | 21 | 20 | 18 | 25 | 39 | 179 |
|  |  |  | CV (%) | 163 | 149 | 156 | 143 | 128 | 100 | 81 | 77 | 96 |
|  |  | + | AVG | 15 | 13 | 11 | 10 | 9 | 6 | 15 | 33 | 147 |
|  |  |  | SD | 25 | 21 | 18 | 16 | 15 | 9 | 16 | 26 | 125 |
|  |  |  | CV (%) | 159 | 160 | 173 | 172 | 171 | 160 | 106 | 80 | 85 |

^1^ not applicable

Supplementary Table S6 (continuation): Average values (AVG, n=5), standard deviations (SD) and coefficients of variation (CV) belonging to the measurements of potential nitrification activities in Icelandic Andosols.

| Location | Andosol class | ATU addition |  | Incubation time (hours) | | | | | | | | |
| --- | --- | --- | --- | --- | --- | --- | --- | --- | --- | --- | --- | --- |
|  |  |  |  | 0 | 3 | 6 | 9 | 12 | 24 | 48 | 72 | 168 |
| 6 | Brown | - | AVG | 0 | 1 | 1 | 1 | 2 | 5 | 9 | 16 | 47 |
|  |  |  | SD | 0 | 2 | 1 | 2 | 4 | 8 | 11 | 17 | 40 |
|  |  |  | CV (%) | n.a.^1^ | 185 | 200 | 165 | 194 | 149 | 123 | 103 | 84 |
|  |  | + | AVG | 0 | 0 | 0 | 0 | 0 | 1 | 1 | 6 | 18 |
|  |  |  | SD | 0 | 0 | 0 | 0 | 0 | 2 | 2 | 7 | 26 |
|  |  |  | CV (%) | n.a. | n.a. | n.a. | n.a. | n.a. | 161 | 161 | 125 | 145 |
| 7 | Brown | - | AVG | 1 | 3 | 3 | 7 | 10 | 22 | 49 | 79 | 260 |
|  |  |  | SD | 1 | 3 | 3 | 5 | 6 | 11 | 21 | 31 | 82 |
|  |  |  | CV (%) | 160 | 109 | 88 | 73 | 65 | 50 | 44 | 39 | 31 |
|  |  | + | AVG | 6 | 7 | 6 | 8 | 9 | 13 | 20 | 28 | 59 |
|  |  |  | SD | 7 | 10 | 10 | 10 | 11 | 11 | 14 | 17 | 23 |
|  |  |  | CV (%) | 124 | 145 | 152 | 123 | 116 | 84 | 67 | 61 | 39 |

^1^ not applicable
